# TALON phase IIIb study: 64 week results of brolucizumab versus aflibercept using treat-and-extend for neovascular age-related macular degeneration

**DOI:** 10.1038/s41433-025-04161-x

**Published:** 2025-12-18

**Authors:** Carl Regillo, Peter K. Kaiser, Peter J. Kertes, Mark Gillies, Tina Maio-Twofoot, David Lawrence, Frank G. Holz

**Affiliations:** 1 Retina Service, Wills Eye Hospital, Thomas Jefferson University, Philadelphia, PA USA; 2 Cole Eye Institute, Cleveland Clinic, Cleveland, OH USA; 3 The John and Liz Tory Eye Centre, Sunnybrook Health Sciences Centre, Toronto, ON Canada; 4 Clinical Ophthalmology and Eye Health, Sydney Eye Hospital, The University of Sydney, Sydney, Australia; 5 Novartis Pharma AG, Basel, Switzerland; 6 Department of Ophthalmology, University of Bonn, Bonn, Germany

**Keywords:** Macular degeneration, Outcomes research

## Abstract

**Objective:**

To compare the efficacy and safety of brolucizumab 6 mg and aflibercept 2 mg in patients with neovascular age-related macular degeneration (nAMD) using an identical 4-week adjustment Treat-and-Extend regimen.

**Methods:**

Patients received brolucizumab (*n* = 366) or aflibercept (*n* = 368) at Weeks 0, 4, 8 and 16, followed by 4-week interval adjustments depending on disease activity (DA) up to a maximum treatment interval of 16 weeks (q16w). After introduction of the urgent safety measure (USM), patients in either arm requiring a 4-week interval were discontinued from study treatment and moved to standard of care (SoC).

**Results:**

At Week 64, more brolucizumab patients had a last treatment interval of q16w with no DA vs aflibercept (28.4% vs 12.2%). In the brolucizumab arm, 22.4%, 26.0% and 23.2% of patients were on treatment intervals of 12, 8 and 4 weeks (on SoC after USM), respectively, compared with 23.9%, 22.0% and 41.8% in the aflibercept arm. The average change in best-corrected visual acuity (letters) from baseline at Weeks 60 and 64 was comparable (brolucizumab: +4.7; aflibercept: +4.9). Average change in central subfield thickness (µm) at Weeks 60 and 64 was −182.9 µm in the brolucizumab arm vs −167.5 µm with aflibercept. Incidence of ocular adverse events (AEs), serious ocular AEs and AEs of special interest in the brolucizumab vs aflibercept arms were 31.1% vs 27.7%, 2.7% vs 0.8%, 6.0% vs 1.6%, respectively.

**Conclusions:**

The Week 64 results in TALON reaffirmed those reported at Week 32, demonstrating extended treatment intervals and an overall favourable benefit/risk profile for brolucizumab in patients with nAMD.

## Introduction

Age-related macular degeneration (AMD) is considered to be the leading cause of vision loss in adults, particularly those over 60 years [1, 2]. It is estimated that by 2040, ~300 million individuals from the developed countries will be affected by AMD [3]. Neovascular AMD (nAMD), an advanced stage of AMD, is characterised by choroidal neovascularisation (CNV) that leads to accumulation of fluid and blood, in the macula, and is typically treated with intravitreal anti-vascular endothelial growth factor (VEGF) injection therapy [1].

In routine clinical practice, anti-VEGF injections are most commonly administered with a loading phase of three monthly injections followed by a treat and extend (T&E) regimen where the treatment interval is tailored to patients’ needs (generally extending in increments of 2 or more weeks) with the goal of maintaining a dry macula, and at the same time, avoiding disease activity recurrence. This approach has been validated in prospective clinical trials with non-inferior visual outcomes to monthly, fixed anti-VEGF injection regimens [4–7]. However, the number of clinic visits still remains relatively high for most patients, and the mean number of injections in T&E clinical trial settings has ranged from 14.1 to 18.6 in the first 2 years of treatment [5–11]. More injections can result in poorer patient compliance, which could eventually result in worse visual outcomes [12–14]. Hence, there is a need for more effective treatments that can lengthen intervals between injections and reduce treatment burden while maintaining functional outcomes [15].

Brolucizumab, a humanised single-chain antibody fragment that has a high affinity for VEGF, is designed for ophthalmic use administered via intravitreal (IVT) injection. The low molecular weight (26 kDa) permits the delivery of more drug per injection, thereby potentially contributing to more effective tissue penetration and increased duration of action [16].

In the pivotal two-year Phase III HAWK and HARRIER studies, brolucizumab 6 mg administered every 12 weeks (q12w; with an option to adjust to every 8 weeks [q8w] if disease activity [DA] was detected) resulted in non-inferior best-corrected visual acuity (BCVA) gains and superior anatomical outcomes compared with aflibercept 2 mg administered with a fixed q8w treatment regimen. Additionally, after receiving 3 monthly loading doses of brolucizumab 6 mg, more than 50% of the treated eyes were maintained on a q12w dosage schedule to Week 48 [17, 18]. Brolucizumab may therefore enable longer injection intervals than aflibercept while providing better anatomical and comparable visual outcomes.

The Phase IIIb TALON study is the first global head-to-head clinical trial to compare brolucizumab and aflibercept using identical T&E regimens allowing interval extensions up to 16 weeks (q16w). At Week 32, the study met both of its co-primary endpoints with brolucizumab achieving superiority to aflibercept in the distribution of last treatment interval with no DA and non-inferiority (margin of 4 letters) to aflibercept for least squares (LS) mean difference in average change in BCVA from baseline at Weeks 28 and 32 in the study eye [19]. Brolucizumab also showed improved anatomical outcomes and demonstrated an overall favourable benefit-risk profile. These results therefore support brolucizumab as a durable treatment option with the potential to dry the retina more effectively and reduce the treatment burden in patients with nAMD. Here, we report the 64-week outcomes from the prospective Phase III TALON study.

## Methods

### Study design and population

TALON (NCT04005352) was a prospective 64-week, randomised, double-masked, multi-centre, active-controlled, two-arm, Phase IIIb study in treatment-naive patients with nAMD. The study was conducted across 20 countries at 118 clinical sites in accordance with the principles of the Declaration of Helsinki. All patients provided written informed consent prior to screening or initiation of any study-related procedures. Study protocols were reviewed and approved by an independent ethics committee or an institutional review board at each participating centre. The study commenced on 25 September 2019, and this report presents the results up to Week 64 (study completion visit) including the secondary efficacy and safety analyses with the last subject last visit dated as 9 September 2022). The comprehensive details of the trial oversight, randomisation, sample size calculations, inclusion and exclusion criteria and the primary objective and endpoints have been published previously [19].

### Randomisation and treatment

**Fig. 1:**
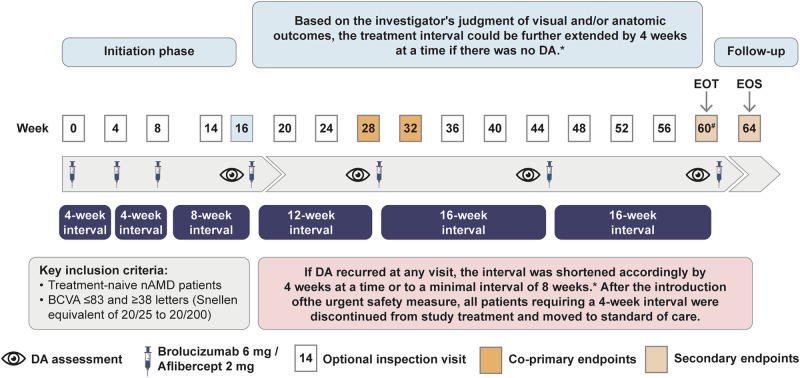
TALON study design. BCVA best-corrected visual acuity, DA disease activity, EOS end of study, EOT end of treatment, nAMD neovascular age-related macular degeneration, q4w every 4 weeks, q8w every 8 weeks, q12w every 12 weeks. At the investigator’s discretion, an inspection visit may have been performed at Week 14, for 8-week treatment interval. If the treatment interval is extended from 8 to 12 weeks, an optional assessment can be performed 10 weeks after the last treatment visit. *The treatment interval could also be maintained if the investigator deemed that the patient would not benefit from injection interval extension. ^#^Week 62, depending on visit schedule.

Patients were randomised 1:1 to receive either brolucizumab 6 mg or aflibercept 2 mg at baseline, Week 4 and Week 8 followed by an 8-week injection interval as shown in Fig. 1 [19]. From Week 16 onwards, based on the masked investigator’s assessment, there was an option to extend the injection interval by 4 weeks at a time if disease activity (DA) was absent to a maximal interval of every 16 weeks. If DA was present at any DA assessment visit, the injection interval was shortened by 4 weeks at a time or to a minimum interval of 4 weeks (however, see the section below on the urgent safety measures [USM]). The injection interval could also be maintained if the investigator deemed that the patient would not benefit from injection interval extension. At the investigator’s discretion, optional inspection visits could be performed 2 weeks prior to an injection whenever the treatment interval was extended. If there was no DA in the study eye at the inspection visit, no treatment was administered, and the next visit and injection took place 2 weeks later. If DA was observed by the masked investigator in the study eye at the inspection visit, the study treatment was administered by the unmasked investigator and the injection interval reduced by 4 weeks.

### Urgent safety measures

Following the 1-year results of the MERLIN study, which showed a higher incidence of intraocular inflammation (IOI) in patients treated every 4 weeks (q4w) with brolucizumab [20], USM were introduced, and the minimum interval between two doses of the study medication during the maintenance phase could not be less than 8 weeks. As per the protocol amendment in response to the USM dated 27 May 2021, and to maintain masking, all patients requiring q4w dosing were discontinued from the study treatment and moved to standard of care by the investigator. In the analysis of the distribution of the last treatment interval presented here, the q4w category represents those subjects who were either on a q4w interval at Week 64 by the time of USM implementation or those who were discontinued from the study treatment after the USM were introduced due to DA on a q8w interval. Investigators were also advised to discontinue the study treatment in patients who developed signs of retinal vasculitis and/or retinal vascular occlusion and to closely monitor those with IOI.

### Study endpoints and assessments

The co-primary endpoints of TALON were the distribution of the last injection interval with no DA up to Week 32 and average change in BCVA from baseline at Weeks 28 and 32, which have been previously reported [19]. The secondary endpoints assessed at Week 64 included distribution of last interval with no DA at Week 64 and average change in BCVA from baseline at Weeks 60 and 64. Other secondary endpoints included (i) BCVA gains of ≥15 letters or BCVA ≥ 84 letters at Week 64, and at last injection visit, (ii) average change from baseline in central subfield thickness (CSFT) as assessed by spectral domain-optical coherence tomography (SD-OCT) using a central reading centre (CRC) at Weeks 60 and 64, (iii) presence of intra-retinal fluid (IRF) and/or sub-retinal fluid (SRF), and sub-retinal pigment epithelium (sub-RPE) fluid in the central subfield as assessed by SD-OCT using a CRC at Weeks 60 and 64, and (iv) incidence of ocular and non-ocular adverse events (AEs) up to Week 64.

### Statistical analyses

Analyses include all randomised subjects who received at least one dose of the study treatment.

Distribution of last treatment interval with no DA up to Week 64 was assessed using a one-sided Wilcoxon test between the brolucizumab versus (vs) aflibercept arms. To assess the average BVCA change from baseline at Weeks 60 and 64 between the brolucizumab vs aflibercept arms, a two-sided 95% confidence interval (CI) for the treatment difference was derived from an analysis of variance (ANOVA) model with treatment arm, baseline BCVA categories (≤54, 55 to ≤73, ≥74 letters) and age categories (<75 years, ≥75 years) as fixed effects. A logistic regression model was used to analyse the number (%) of patients with occurrence of BCVA improvements of ≥15 letters from baseline as well as BCVA ≥ 84 letters or ≥69 letters at Week 64. Change from baseline in CSFT (μm) for the average at Weeks 60 and 64 for the study eye was analysed using an ANOVA model with baseline CSFT (<400 μm, ≥400 μm) and age as categorical variables (<75 years, ≥75 years) and treatment as fixed effect.

All safety evaluations were descriptive. The AEs reported in the Week 64 data were coded using the Medical Dictionary for Regulatory Activities version 25.0. Full details of statistical analyses performed in TALON have also been published previously [19].

## Results

### Patient population/subject disposition

In total, 734 patients were randomised and treated in the TALON study with 366 patients in the brolucizumab arm and 368 patients in the aflibercept arm. Patient disposition details are shown in Supplementary Fig. 1. Demographic and baseline characteristics were comparable between treatment arms (Supplementary Table 1) and have been published previously [19]. Overall, 87 patients (23.6%) in the brolucizumab arm and 116 patients (31.4%) in the aflibercept arm discontinued the study treatment prior to or at Week 64. The most common reason for study treatment discontinuation in the brolucizumab vs aflibercept arms was sponsor request (10.3% vs 22.5%), due to the protocol amendment following the USM (i.e. patients requiring q4w treatment intervals discontinued study treatment but remained in the study).

### Distribution of last treatment interval with no DA at Week 64

**Fig. 2:**
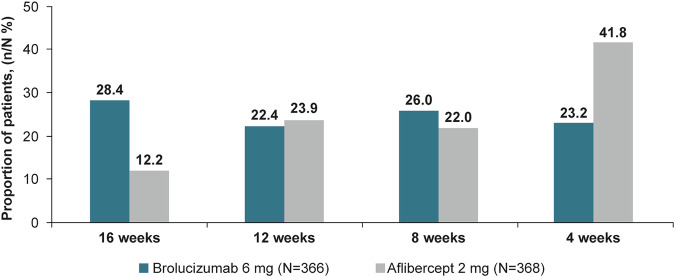
Proportion of patients with the last interval with no DA* (n/N %) at Week 64. Pre-specified secondary endpoint. *DA* disease activity, *n* the number of participants at 4-/8-/12-/16-week intervals as the last interval with no DA, *N* the number of participants in the full analysis set per treatment arm, *q4w* every 4 weeks, *q8w* every 8 weeks, *q12w* every 12 weeks, *q16w* every 16 weeks. DA is as assessed by an investigator. If the duration of the last interval falls within the following ranges of (q4w, q8w) or (q8w, q12w) or (q12w, q16w) or ≥q16w, then the floor value of these ranges i.e. q4w, q8w, or q12w, q16w, respectively, are used for imputation.

In the analysis of this co-primary endpoint at Week 32, brolucizumab 6 mg was superior to aflibercept in the distribution of last interval with no DA [19]. At Week 64, more brolucizumab patients had a last treatment interval of q16w with no DA, whereas more aflibercept patients demonstrated a q4w interval need. In the brolucizumab arm, 28.4%, 22.4%, 26.0% and 23.2% of patients were on treatment intervals of 16, 12, 8 and 4 weeks, respectively, compared with 12.2%, 23.9%, 22.0% and 41.8% in the aflibercept arm, respectively; *P* < 0.0001 (Fig. 2).

### Average change in BCVA from baseline to Weeks 60 and 64

**Fig. 3:**
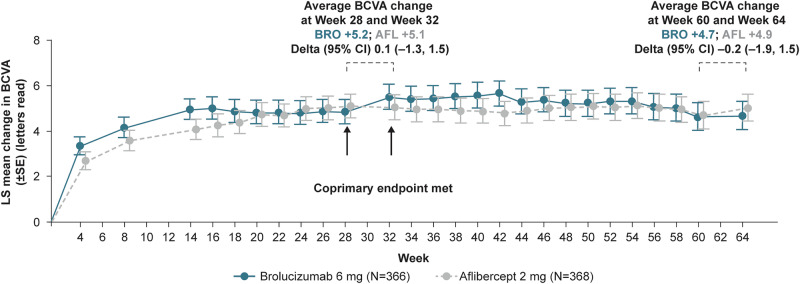
Mean change in BCVA from baseline to Week 64. Pre-specified secondary endpoint. AFL aflibercept, ANOVA Analysis of variance, BCVA best-corrected visual acuity, BRO brolucizumab, CI confidence interval, LOCF Last Observation Carried Forward, LS least squares, SE standard error. LOCF. ANOVA model with treatment arm (factor), baseline BCVA categories (≤54, 55 to ≤73, ≥74 letters), and age categories (<75 years, ≥75 years) as fixed effects. BCVA values collected after the start of an alternative treatment were replaced by the last value prior to start of alternative treatment. If Week 60 BCVA value is not available, Week 62 BCVA is used; otherwise LOCF is applied. The primary objective was met upon significance of both non-inferiority of average change in BCVA from baseline at Weeks 28 and 32 and superiority of distribution of the last interval without DA up to Week 32.

In the analysis of this co-primary endpoint, brolucizumab was non-inferior to aflibercept in average change in BCVA from baseline at Weeks 28 and 32 [19]. BCVA gains achieved with brolucizumab 6 mg were maintained through Week 64 and were comparable to aflibercept. The average change in BCVA from baseline at Weeks 60 and 64 was +4.7 letters in the brolucizumab arm compared with +4.9 letters in the aflibercept arm, with a treatment difference of −0.2 letters (95% CI: −1.9, 1.5) (Fig. 3).

Similar to Week 32, the proportions of patients who gained ≥15 letters from baseline or who had ≥84 letters at Week 64 were comparable between the brolucizumab vs aflibercept arms (24.3% vs 24.7%) (Supplementary Fig. 2). The proportion of patients with occurrence of BCVA ≥ 69 letters was 65.6% in the brolucizumab arm and 59.8% in the aflibercept arm at Week 64 as shown in Supplementary Fig. 3.

### Structural outcomes

**Fig. 4:**
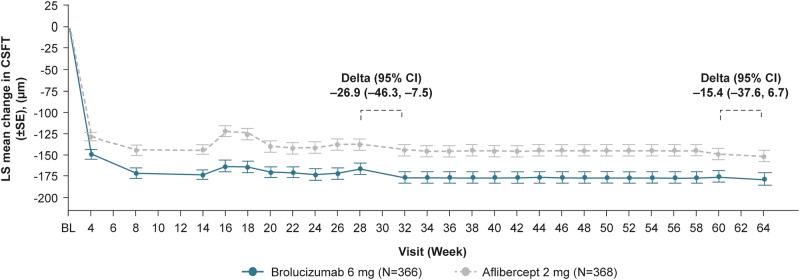
Line plot of mean change in CSFT from baseline by visit up to Week 64. Secondary endpoint. ANOVA Analysis of variance, CI confidence interval, CSFT central subfield thickness, LOCF last observation carried forward, LS least squares, SE standard error. ANOVA baseline CSFT categories (<400 μm, ≥400 μm), age categories (<75 years, ≥75 years) and treatment as fixed effects. If Week 28/60 CSFT value is not available, Week 30/62 CSFT is used, otherwise LOCF is used.

Brolucizumab achieved greater reductions vs aflibercept in the average change in CSFT from baseline at Weeks 60 and 64 consistent with Weeks 28 and 32. The LS mean difference was ˗15.4 µm (95% CI: −37.6, +6.7; brolucizumab −182.9 µm vs aflibercept −167.5 µm) (Fig. 4).

Fewer brolucizumab-treated patients had IRF and/or SRF vs aflibercept-treated patients at Week 60 (22.3% vs 28.3%) and Week 64 (26.6% vs 34.4%) and these patterns were comparable with Week 32 results. A similar trend was also observed for sub-RPE fluid for brolucizumab vs aflibercept arms at Week 60 (10.0% vs 12.7%) and Week 64 (12.5% vs 17.8%) (Supplementary Fig. 4).

### Safety outcomes

The overall safety profile of brolucizumab and aflibercept up to Week 64 is shown in Table 1. Up to Week 64, 35.5% of the patients in the brolucizumab arm and 33.7% of the patients in the aflibercept arm experienced at least one ocular AE. The most frequently reported ocular AEs by preferred term were conjunctival haemorrhage (6.3% vs 3.5%), visual acuity reduced (4.9% vs 5.2%) and eye pain (4.6% vs 3.5%) in the brolucizumab arm vs the aflibercept arm, respectively (Supplementary Table 2). The incidence of non-ocular AEs reported by preferred term was comparable between the brolucizumab and aflibercept treatment arms (49.7% vs 50.3%) and is shown in (Supplementary Table 3).

**Table 1:** Overall safety profile of brolucizumab and aflibercept up to Week 64.

Adverse event	Brolucizumab 6 mg (*N* = 366), *n* (%)	Aflibercept 2 mg (*N* = 368), *n* (%)
Patients with ≥ 1 AE		
*Ocular in the study eye*	130 (35.5)	124 (33.7)
*Non-ocular*	182 (49.7)	185 (50.3)
Patients with ≥ 1 serious AE		
*Ocular in the study eye*	11 (3.0)	3 (0.8)
*Non-ocular*	49 (13.4)	50 (13.6)
Adverse events leading to study treatment discontinuation		
*Ocular in the study eye*	18 (4.9)	3 (0.8)
*Non-ocular*	0	3 (0.8)
Deaths	4 (1.1)	2 (0.5)
Adverse event of special interest^a^		
*Ocular in the study eye*	22 (6.0)	6 (1.6)

*AEs* adverse events, *n* number present count of patients, *N* number of patients in the analysis set.

^a^ IOI including retinal vasculitis, endophthalmitis and retinal vascular occlusions. Safety analysis set. AEs are reported from the on-treatment period, i.e. events occurring from the date of first administration of the study treatment to 30 days after the last administration of study treatment or end of study, whichever is the latest. MedDRA Version 25.0 has been used for the reporting of AEs.

Serious ocular AEs were reported in 14 patients up to Week 64: 11 (3.0%) in the brolucizumab arm and three (0.8%) in the aflibercept arm (Supplementary Table 2). Most occurred in the first 32 weeks of the study, and the incidence of serious ocular AEs remained low from Week 32 to 64 in both the brolucizumab (two patients) and aflibercept (one patient) arms. Of the 11 serious ocular AEs reported in the brolucizumab arm up to Week 64, eight were AEs of special interest (AESIs), including IOI (with retinal vasculitis), retinal vascular occlusion and endophthalmitis, and eight of the 11 patients discontinued the study treatment due to a serious ocular AE. In the aflibercept arm, one of the three reported serious ocular AEs led to study treatment discontinuation. Non-ocular SAEs were comparable in the brolucizumab and aflibercept arms (13.4% vs 13.6%, respectively).

A total of 28 patients (22 [6.0%] in the brolucizumab arm and six [1.6%] in the aflibercept arm) reported at least one AESI up to Week 64, and the majority occurred in the initial 32-week period (20 of the 22 in the brolucizumab and four of the six in the aflibercept arm). From Week 32 to 64, two new cases of AESIs were reported in each arm (brolucizumab: one patient with uveitis and one with iritis; aflibercept: one patient with iridocyclitis and one with uveitis). Overall, up to Week 64, IOI (including retinal vasculitis) was noted more frequently in the brolucizumab arm vs the aflibercept arm (16 [4.4%] vs five [1.4%], respectively). Infectious endophthalmitis was reported in one (0.3%) vs zero (0.0%), and retinal vascular occlusion events in five (1.4%) vs one patient (0.3%) in the brolucizumab and aflibercept arms, respectively (Supplementary Table 4).

Similar to Week 32, the proportion of all patients who lost ≥15 letters from baseline at Week 64 was comparable between the brolucizumab (5.5%) and aflibercept (5.3%) arms (Supplementary Table 5). In the patients who experienced AESIs during the course of the study, one of the 22 in the brolucizumab arm and zero of the six in the aflibercept arm lost ≥15 letters from baseline at Week 64 (Supplementary Table 6). A further eight patients (six in the brolucizumab arm and two in the aflibercept arm) with AESI initially lost ≥15 letters from baseline but their vision improved subsequently such that they no longer fell into this category at Week 64. Two of these cases of vision loss (one in each arm) occurred between Week 32 and Week 64

A total of six deaths were reported up to Week 64, four (1.1%) in the brolucizumab arm (cardiac disorders, COVID-19 infection and pneumonia) and two (0.5%) in the aflibercept arm (acute respiratory failure and peripheral artery aneurysm rupture). None of the reported deaths were deemed by the investigator to be related to the study treatments.

## Discussion

TALON is the first study to compare brolucizumab and aflibercept using matched T&E regimens allowing intervals for retreatment up to q16w. The Week 64 results are consistent with the Week 32 results, with more brolucizumab-treated patients achieving longer treatment intervals without DA while maintaining comparable visual gains, and with better structural outcomes compared to those treated with aflibercept. AESIs occurred in accordance with what has been previously reported, supporting the overall favourable benefit/risk profile of brolucizumab.

In routine clinical practice and in many real-world studies, visual outcomes associated with the use of anti-VEGFs for nAMD are less favourable when compared to the clinical trials owing, at least in part, to a significant treatment burden for both physicians and patients leading to lower injection rates [12–14]. T&E regimens are commonly used in routine clinical practice to treat patients with nAMD, to enhance the visual outcomes while reducing the overall treatment and visit burden. In the TALON primary analysis, brolucizumab 6 mg achieved superiority to aflibercept in the distribution of last interval with no DA at Week 32 [19], and at Week 64 more patients in the brolucizumab 6-mg arm were treated with a 16-week interval. More patients in the brolucizumab arm were on extended treatment regimens (q12w and q16w) at Week 64 compared to the aflibercept arm, further demonstrating the extended and sustained disease control with brolucizumab. The findings from the 64-week results favour brolucizumab use in a more efficient T&E approach with longer treatment intervals, and thereby, reduced treatment burden.

Mean BCVA gains at Week 32 were maintained to Week 64. In addition, in comparison to the aflibercept arm, the brolucizumab arm consistently showed a greater numerical reduction in LS mean change from baseline in CSFT at all study visits to Week 64 with, for example, a treatment difference at Week 32 of −26.9 µm [19]. With regard to the overall anatomical results, brolucizumab 6 mg showed greater improvements than aflibercept 2 mg, implying a more effective and long-lasting regulation of DA.

The incidence of AESIs was higher in the brolucizumab arm vs aflibercept arm. At Week 64, a total of 28 patients (22 patients [6.0%] in the brolucizumab arm and six patients [1.6%] in the aflibercept arm) had at least one AESI reported for the study eye. Notably, most AESIs were reported in the first 32-week period (20 patients [5.5%] in the brolucizumab arm and four patients [1.1%] in the aflibercept arm). This incidence and timings are consistent with the previously reported AESIs associated with brolucizumab, which were affirmed in the year 2020 after a post hoc evaluation of the IOI-related AEs in the Phase III HAWK and HARRIER studies [21]. Immunogenicity has been considered as a prerequisite to the development of retinal vasculitis/retinal vascular occlusion [22, 23]; however, further research is needed to fully understand the mechanisms of immunogenicity against brolucizumab. Evidence-based recommendations have been developed by several groups of ophthalmologists highlighting that monitoring and vigilance for symptoms of IOI during treatment plays a crucial role in the care of patients treated with brolucizumab [21, 24–30]. Prior to injection, the eye should be thoroughly inspected for inflammation, and patients should be advised to report any change in vision or symptoms of IOI (including retinal vasculitis) and/or retinal vascular occlusion immediately so that the adverse events can be promptly managed [24–30]. Up to Week 64, a total of nine patients (seven in the brolucizumab arm and two in the aflibercept arm) initially lost ≥15 letters due to an AESI. The majority of these occurred prior to Week 32 (six in the brolucizumab arm and one in the aflibercept arm). Vision subsequently improved in all patients apart from one in the brolucizumab arm. This suggests that increased awareness, vigilance and prompt treatment might have prevented progression to a more severe, irreversible event.

The strengths of the TALON study include that it is the first head-to-head study to compare brolucizumab with aflibercept for the treatment of nAMD using an identical T&E regimen, which closely mimics routine clinical practice. The limitations of the study include the introduction of the USM, which meant that patients on q4w could not be continued on study treatment and may have been eligible for subsequent extension. In addition, 4-week interval extensions did not allow for further fine-tuning of the treatment interval as some patients may have tolerated a further 2-week extension. This study presents data only up to Week 64; hence, we cannot comment on the long-term durability and safety of brolucizumab. The 56-week TALON extension study will provide more long-term efficacy and safety data regarding the use of brolucizumab in a T&E regimen up to an interval of 20 weeks and for a total of 120 weeks of treatment.

In conclusion, the overall efficacy results from Week 64 confirmed the results from Week 32. The safety profile of brolucizumab was consistent with the previously established profile of brolucizumab in the treatment of nAMD and no new safety concerns were identified. The 64-week results from the TALON study thus suggest brolucizumab as a long-lasting therapeutic option with the potential to improve retinal drying and reduce treatment burden in patients with nAMD.

Supplementary material is available at Eye’s website.

## Summary

### What was known before

- Various clinical trials have demonstrated that anti-vascular endothelial growth factor treatments are effective for treating neovascular age-related macular degeneration (nAMD), but this is associated with a significant treatment burden resulting in reduced compliance and eventually poor visual outcomes as visits and injections are missed.
- There is still a need for highly effective treatments that can prolong intervals between injections and reduce treatment burden without compromising vision outcomes.

### What this study adds

- The TALON Phase IIIb study is the first prospective, head-to-head clinical study, which compared brolucizumab and aflibercept using identical Treat & Extend regimens allowing interval extensions up to 16 weeks.
- This study supports brolucizumab as a durable treatment option with the potential to dry the retina more effectively and reduce treatment burden in patients with nAMD.

## Supplementary information

SF1 Patient disposition (all enrolled patients) - CONSORT flow diagram

SF2 Number (%) patients of who gained ≥15 letters in BCVA from baseline

SF3 Number (%) patients with occurrence of BCVA ≥69 letters at Weeks 32 and 64

SF4 Proportion of patients with IRF and/or SRF and sub-RPE fluid at Weeks 28 and 32, and at Weeks 60 and 64

ST1 Patient demographics and baseline disease characteristics

ST2 Ocular adverse events (≥1% in any treatment arm) and serious ocular adverse events by preferred term for the study eye

ST3 Non-ocular adverse events (≥2% in any treatment arm) by preferred term

ST4 Adverse events of special interest (IOI including retinal vasculitis, endophthalmitis and retinal vascular occlusions) through Week 64

ST5 Number (%) of patients who lost ≥15 letters in BCVA from baseline at Week 64 for the study eye

ST6 Number (%) of patients with adverse events of special interest who lost ≥15 letters in BCVA from baseline at Week 64 for the study eye

## Data Availability

All data generated or analysed during this study are included in this published article [and its supplementary information files].
