# Supplementary material for: TALON phase IIIb study: 64 week results of brolucizumab versus aflibercept using treat-and-extend for neovascular age-related macular degeneration
Source: Eye (Lond). 2025 Dec 18;40(3):369–75. doi: 10.1038/s41433-025-04161-x (PMC12881385; doi:10.1038/s41433-025-04161-x)
Supplement: Supplementary file 1 — SF1 Patient disposition (all enrolled patients) - CONSORT flow diagram [file 41433_2025_4161_MOESM1_ESM.pdf]

Supplementary Fig. 1 Patient disposition (all enrolled patients) - CONSORT flow diagram

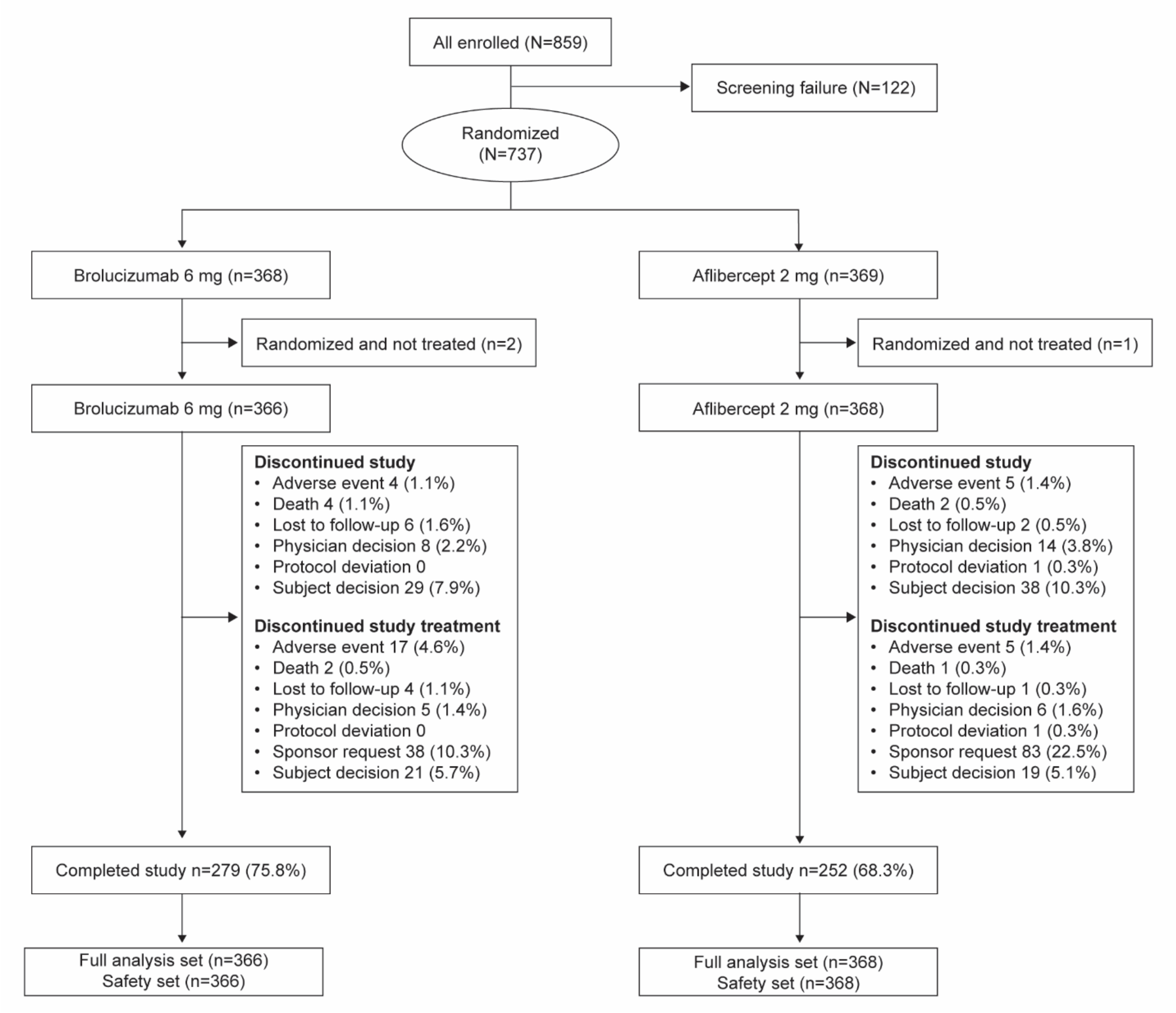

CRF case report form.

Percentages (%) are calculated based on "n" from "all randomised" category

The reason for discontinuation as given by the investigator in the CRF.
