# Supplementary material for: TALON phase IIIb study: 64 week results of brolucizumab versus aflibercept using treat-and-extend for neovascular age-related macular degeneration
Source: Eye (Lond). 2025 Dec 18;40(3):369–75. doi: 10.1038/s41433-025-04161-x (PMC12881385; doi:10.1038/s41433-025-04161-x)
Supplement: Supplementary file 3 — SF3 Number (%) patients with occurrence of BCVA ≥69 letters at Weeks 32 and 64 [file 41433_2025_4161_MOESM3_ESM.pdf]

Supplementary Fig. 3 Number (%) patients with occurrence of BCVA ≥69 letters at Weeks 32 and 64

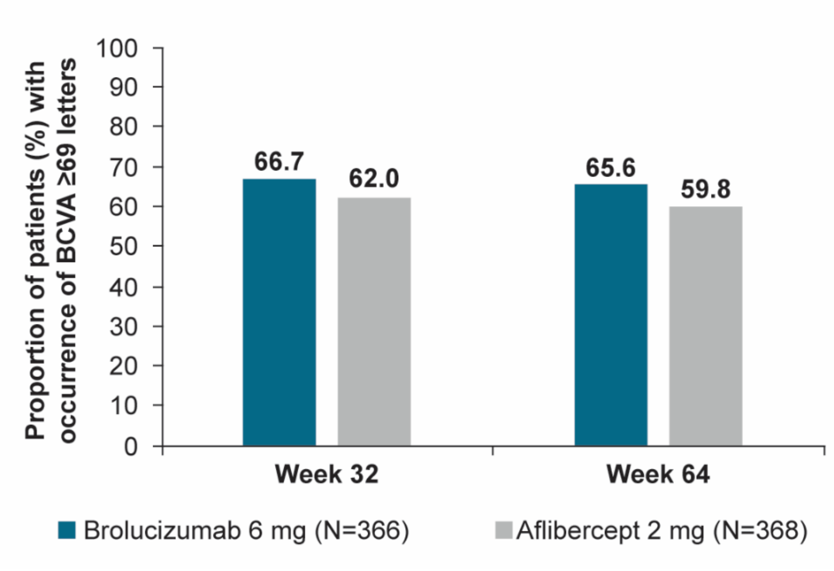

BCVA best-corrected visual acuity, *LOCF* Last Observation Carried Forward.

LOCF is applied.

BCVA values collected after the start of an alternative treatment were replaced by the last value on/prior to start of alternative treatment.
