# Supplementary material for: TALON phase IIIb study: 64 week results of brolucizumab versus aflibercept using treat-and-extend for neovascular age-related macular degeneration
Source: Eye (Lond). 2025 Dec 18;40(3):369–75. doi: 10.1038/s41433-025-04161-x (PMC12881385; doi:10.1038/s41433-025-04161-x)
Supplement: Supplementary file 4 — SF4 Proportion of patients with IRF and/or SRF and sub-RPE fluid at Weeks 28 and 32, and at Weeks 60 and 64 [file 41433_2025_4161_MOESM4_ESM.pdf]

**Supplementary Fig. 4** Proportion of patients with IRF and/or SRF and sub-RPE fluid at Weeks 28 and 32, and at Weeks 60 and 64

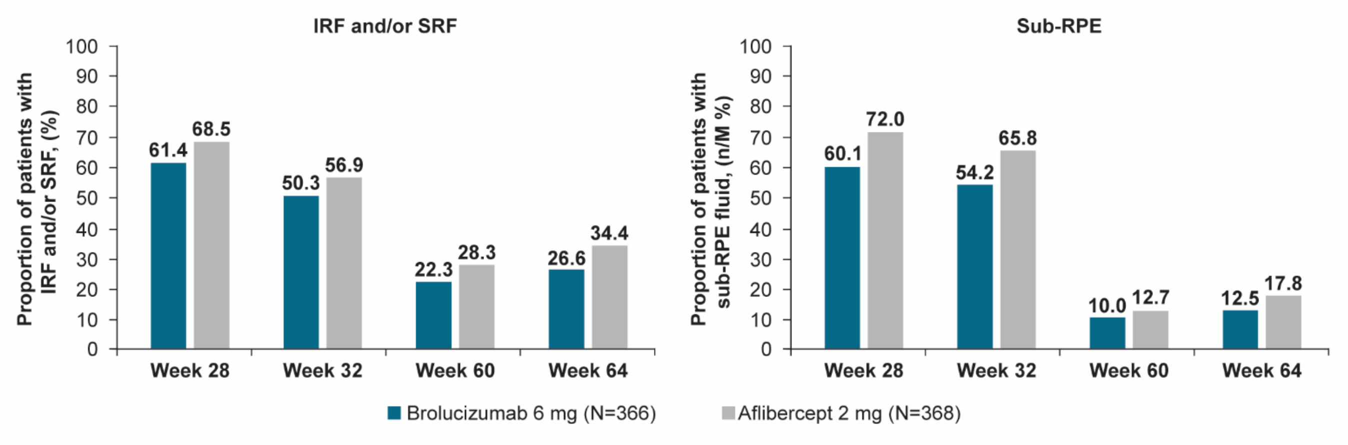


*IRF* intraretinal fluid, *RPE* retinal pigment epithelium, *SRF* subretinal fluid.

IRF/SRF and sub-RPE values collected after the start of an alternative treatment in the study eye are replaced by the last value on/before to start of this alternative treatment.

If Week 28/60 data were not available, then Week 30/62 data were used instead of Week 28/60.
