# Supplementary material for: TALON phase IIIb study: 64 week results of brolucizumab versus aflibercept using treat-and-extend for neovascular age-related macular degeneration
Source: Eye (Lond). 2025 Dec 18;40(3):369–75. doi: 10.1038/s41433-025-04161-x (PMC12881385; doi:10.1038/s41433-025-04161-x)
Supplement: Supplementary file 5 — ST1 Patient demographics and baseline disease characteristics [file 41433_2025_4161_MOESM5_ESM.pdf]

Supplementary Table 1. Patient demographics and baseline disease characteristics

Characteristics	Brolucizumab 6 mg (N = 366)	Aflibercept 2 mg (N = 368)
**Age (years), mean (SD)**	75.5 (7.85)	75.5 (8.41)
<75 years, m (%)	161 (44.0)	151 (41.0)
≥75 years, m (%)	205 (56.0)	217 (59.0)
**Sex, m (%)**		
Male	150 (41.0)	164 (44.6)
Female	216 (59.0)	204 (55.4)
**Race, m (%)**		
White	310 (84.7)	312 (84.8)
Black or African American	1 (0.3)	1 (0.3)
Asian	55 (15.0)	55 (14.9)
Chinese	14 (3.8)	10 (2.7)
Indian	4 (1.1)	1 (0.3)
Korean	25 (6.8)	28 (7.6)
**BCVA (letters), mean (SD)**	63.88 (12.13)	63.50 (11.98)
**BCVA group, n (%)**		
≤54 letters	75 (20.7)	94 (25.6)
55 to ≤73 letters	200 (55.1)	188 (51.2)
≥74 letters	88 (24.2)	85 (23.2)
**CSFT (μm), mean (SD)**	443.75 (164.48)	466.97 (163.29)
**CSFT group, n (%)**		
<400 μm	174 (48.3)	140 (38.8)
≥400 μm	186 (51.7)	221 (61.2)
**Type of CNV, n (%)**		
Predominantly classic	23 (6.5)	32 (8.8)
Minimally classic	47 (13.2)	55 (15.2)
Occult	190 (53.4)	193 (53.3)
Fibrovascular PED	114 (32.0)	121 (33.4)
Serious PED	8 (2.2)	9 (2.5)
Late leakage	68 (19.1)	63 (17.4)
**CNV lesion categories, n (%)**		
Type 1	158 (48.8)	166 (50.5)
Type 2	125 (38.6)	116 (35.3)
Type 3	40 (12.3)	46 (14.0)
Cannot grade	1 (0.3)	0
**Subretinal fluid, n (%), present**	282 (79.2)	272 (75.6)
**Intraretinal fluid/cyst, n (%), present**	165 (46.1)	174 (48.9)
**Sub-RPE fluid, n (%), present**	218 (61.2)	234 (64.8)

BCVA best-corrected visual acuity, *CSFT* central subfield thickness, *CNV* choroidal neovascularisation, *m* number of patients with assessment meeting the criterion for the given categorical variables, *N* number of patients in full analysis set, *n* number of patients with an assessment, *RPE* retinal pigment epithelium, *SD* standard deviation.
Percentages (%) are calculated based on n.
Occult is considered present if at least one of the three sub-types (fibrovascular PED, serious PED and late leakage) is present.
"Predominantly classic" category includes both "predominantly classic" and "pure classic" sub-categories.
