# Supplementary material for: TALON phase IIIb study: 64 week results of brolucizumab versus aflibercept using treat-and-extend for neovascular age-related macular degeneration
Source: Eye (Lond). 2025 Dec 18;40(3):369–75. doi: 10.1038/s41433-025-04161-x (PMC12881385; doi:10.1038/s41433-025-04161-x)
Supplement: Supplementary file 6 — ST2 Ocular adverse events (≥1% in any treatment arm) and serious ocular adverse events by preferred term for the study eye [file 41433_2025_4161_MOESM6_ESM.pdf]

Supplementary Table 2. Ocular adverse events (≥1% in any treatment arm) and serious ocular adverse events by preferred term for the study eye

**Preferred term**	**Brolucizumab 6 mg (N = 366), n (%)**	**Aflibercept 2 mg (N = 368), n (%)**
**Ocular adverse events**		
Number of patients with at least one event	130 (35.5)	124 (33.7)
Conjunctival haemorrhage	23 (6.3)	13 (3.5)
Eye pain	17 (4.6)	13 (3.5)
Visual acuity reduced	18 (4.9)	19 (5.2)
Vitreous floaters	14 (3.8)	6 (1.6)
Vitreous detachment	11 (3.0)	3 (0.8)
Cataract	5 (1.4)	7 (1.9)
Retinal pigment epithelial tear	5 (1.4)	4 (1.1)
Intra-ocular injection complication	6 (1.6)	3 (0.8)
Retinal artery occlusion	4 (1.1)	0
Foreign body sensation in eyes	5 (1.4)	8 (2.2)
Intraocular pressure increased	5 (1.4)	11 (3.0)
Subretinal fluid	5 (1.4)	11 (3.0)
Dry eye	8 (2.2)	18 (4.9)
Macular oedema	3 (0.8)	4 (1.1)
Retinal haemorrhage	4 (1.1)	7 (1.9)
Detachment of retinal pigment epithelium	0	4 (1.1)
Conjunctivitis	5 (1.4)	5 (1.4)
Posterior capsule opacification	3 (0.8)	5 (1.4)
Neovascular age-related macular degeneration	3 (0.8)	4 (1.1)
Hordeolum	4 (1.1)	2 (0.5)
Eye pruritus	4 (1.1)	1 (0.3)
Retinal depigmentation	1 (0.3)	4 (1.1)
Retinal oedema	1 (0.3)	4 (1.1)
Uveitis	4 (1.1)	1 (0.3)
Vision blurred	4 (1.1)	1 (0.3)
Subretinal fibrosis	4 (1.1)	0
**Serious ocular adverse events**		
Number of patients with at least one event	11 (3.0)	3 (0.8)
Uveitis	3 (0.8)	0
Endophthalmitis	1 (0.3)	0
Eye inflammation	1 (0.3)	0
Iridocyclitis	1 (0.3)	1 (0.3)
Retinal artery occlusion	1 (0.3)	0
Retinal vascular occlusion	1 (0.3)	0

Visual acuity reduced	1 (0.3)	0
Glaucoma	0	1 (0.3)
Intraocular pressure increased	0	1 (0.3)
Retinal vein occlusion	0	1 (0.3)
Macular hole	1 (0.3)	0
Ocular discomfort	1 (0.3)	0

Safety analysis set.
AEs adverse events, *n* number of patients with at least one AE for the specific category, *N* number of patients in analysis set.
A subject with multiple occurrences of an AE for a preferred term is counted only once in each specific category.
AEs are reported from the on-treatment period, i.e. events occurring from the date of first administration of the study treatment to 30 days after the last administration of study treatment or end of study, whichever is the latest.
MedDRA Version 25.0 has been used for the reporting of AEs.
