# Supplementary material for: TALON phase IIIb study: 64 week results of brolucizumab versus aflibercept using treat-and-extend for neovascular age-related macular degeneration
Source: Eye (Lond). 2025 Dec 18;40(3):369–75. doi: 10.1038/s41433-025-04161-x (PMC12881385; doi:10.1038/s41433-025-04161-x)
Supplement: Supplementary file 7 — ST3 Non-ocular adverse events (≥2% in any treatment arm) by preferred term [file 41433_2025_4161_MOESM7_ESM.pdf]

Supplementary Table 3. Non-ocular adverse events (≥2% in any treatment arm) by preferred term

**Preferred term**	**Brolucizumab 6 mg (N = 366), n (%)**	**Aflibercept 2 mg (N = 368), n (%)**
Number of patients with at least one event	182 (49.7)	185 (50.3)
Hypertension	18 (4.9)	17 (4.6)
COVID-19	11 (3.0)	16 (4.3)
Nasopharyngitis	12 (3.3)	11 (3.0)
Headache	10 (2.7)	11 (3.0)
Urinary tract infection	11 (3.0)	10 (2.7)
Fall	6 (1.6)	11 (3.0)
Back pain	8 (2.2)	8 (2.2)

Safety analysis set.
AE adverse event, *n* number of patients with at least one AE for the specific category, *COVID-19* coronavirus disease-2019, *N* number of patients in analysis set.
A subject with multiple occurrences of an AE for a preferred term is counted only once in each specific category.
MedDRA Version 25.0 has been used for the reporting of AEs.
