# Supplementary material for: TALON phase IIIb study: 64 week results of brolucizumab versus aflibercept using treat-and-extend for neovascular age-related macular degeneration
Source: Eye (Lond). 2025 Dec 18;40(3):369–75. doi: 10.1038/s41433-025-04161-x (PMC12881385; doi:10.1038/s41433-025-04161-x)
Supplement: Supplementary file 8 — ST4 Adverse events of special interest (IOI including retinal vasculitis, endophthalmitis and retinal vascular occlusions) through Week 64 [file 41433_2025_4161_MOESM8_ESM.pdf]

Supplementary Table 4. Adverse events of special interest (IOI including retinal vasculitis, endophthalmitis and retinal vascular occlusions) through Week 64

	**Brolucizumab 6 mg (N = 366), n (%)**	**Aflibercept 2 mg (N = 368), n (%)**
**Patients with at least one event**	22 (6.0)	6 (1.6)
**Patients with at least one endophthalmitis event**	1 (0.3)	0
**Patients with at least one IOI event**	16 (4.4)	5 (1.4)
Iridocyclitis	3 (0.8)	2 (0.5)
Uveitis	4 (1.1)	1 (0.3)
Eye inflammation	2 (0.5)	0
Retinal occlusive vasculitis	2 (0.5)	0
Vitritis	2 (0.5)	0
Anterior chamber inflammation	1 (0.3)	1 (0.3)
Retinal vasculitis	1 (0.3)	0
Anterior chamber cell	0	1 (0.3)
Anterior chamber flare	0	1 (0.3)
Vitreous haze	0	1 (0.3)
Iritis	1 (0.3)	0
**Patients with at least one retinal vascular occlusion event**	5 (1.4)	1 (0.3)
Retinal artery occlusion	4 (1.1)	0
Retinal vascular occlusion	1 (0.3)	0
Retinal vein occlusion	0	1 (0.3)

Safety analysis set.
AE adverse event, *IOI* intraocular inflammation, *n* number of patients with at least one AE for the specific category, *N* number of patients in analysis set.
A subject with multiple occurrences of an AE for a preferred term is counted only once in each specific category.
MedDRA Version 25.0 has been used for the reporting of AEs.
