# Supplementary material for: TALON phase IIIb study: 64 week results of brolucizumab versus aflibercept using treat-and-extend for neovascular age-related macular degeneration
Source: Eye (Lond). 2025 Dec 18;40(3):369–75. doi: 10.1038/s41433-025-04161-x (PMC12881385; doi:10.1038/s41433-025-04161-x)
Supplement: Supplementary file 9 — ST5 Number (%) of patients who lost ≥15 letters in BCVA from baseline at Week 64 for the study eye [file 41433_2025_4161_MOESM9_ESM.pdf]

Supplementary Table 5. Number (%) of patients who lost ≥15 letters in BCVA from baseline at Week 64 for the study eye

	**n/M (%)**
Brolucizumab 6 mg (N = 366)	15/273 (5.5)
Aflibercept 2 mg (N = 368)	13/244 (5.3)

Safety analysis set.
BCVA best-corrected visual acuity, *M* number of patients with BCVA assessment, *n* number of patients with BCVA loss of at least 15 letters from the baseline, *N* number of patients in analysis set.
