# Supplementary material for: TALON phase IIIb study: 64 week results of brolucizumab versus aflibercept using treat-and-extend for neovascular age-related macular degeneration
Source: Eye (Lond). 2025 Dec 18;40(3):369–75. doi: 10.1038/s41433-025-04161-x (PMC12881385; doi:10.1038/s41433-025-04161-x)
Supplement: Supplementary file 10 — ST6 Number (%) of patients with adverse events of special interest who lost ≥15 letters in BCVA from baseline at Week 64 for the study eye [file 41433_2025_4161_MOESM10_ESM.pdf]

Supplementary Table 6. Number (%) of patients with adverse events of special interest who lost ≥15 letters in BCVA from baseline at Week 64 for the study eye

	**n/M (%)**
Brolucizumab 6 mg (N = 22)	1/22 (9.1)
Aflibercept 2 mg (N = 6)	0/6 (0.0)

Safety analysis set.
AEs adverse events, *AESIs* adverse event of special interest, *BCVA* best-corrected visual acuity, *n* number of patients ≥15 letters loss in BCVA at Week 64 or last visit in a subset with AESIs, *N* number of patients in analysis set with AESIs, *M* number of patients with BCVA at Week 64 or last visit in subset with AESIs.
